# The ABO blood group and *Plasmodium falciparum *malaria in Awash, Metehara and Ziway areas, Ethiopia

**DOI:** 10.1186/1475-2875-9-280

**Published:** 2010-10-12

**Authors:** Zinaye Tekeste, Beyene Petros

**Affiliations:** 1 Department of Biology, Addis Ababa University, P.O. Box 1176, Addis Ababa, Ethiopia; 2 Department of Microbiology and Parasitology, Gondar University, Gondar, Ethiopia

## Abstract

**Background:**

The virulence of *Plasmodium falciparum *is associated with the capacity of the infected red blood cell (iRBC) to adhere to uninfected RBCs, a process known as rosetting, which has been linked to the occurrence of severe malaria. The present study was carried out in three Ethiopian malaria endemic localities to investigate the relationship between blood group type and severe disease in falciparum malaria.

**Methods:**

A total of 210 cases of malaria (70 severe and 140 uncomplicated) and 190 healthy controls participated in the study. Patients with at least one of the severe malaria syndromes (cerebral malaria, severe anaemia and circulatory collapse) were considered as severe malaria cases.

**Results:**

In the severe malaria category, there were 25 (35.7%), 15 (21.4%), 14 (20%) and 16 (22.9%) blood group A, B, AB and O patients, respectively. Blood group O was the dominant blood type in both uncomplicated malaria (45.7%) and healthy controls (41.6%). A case of severe malaria was almost twice as likely to be of type A as to be of type O (odds ratio (OR) 0.42, 95% confidence interval (CI) 0.20-0.88, P = 0.019), and more than twice as likely to be of type B as to be of type O (OR 0.38, 95% CI 0.16-0.89, P = 0.02). Furthermore, individuals with severe malaria were about six fold less likely to be of O as to be of type AB (OR 0.19, 95% CI 0.07-0.51, P = 0.0005).

**Conclusion:**

The study revealed that on the basis of the three criteria (cerebral malaria, severe anaemia and circulatory collapse) used to determine severity in *P. falciparum *malaria, patients with blood group O, which is less prone to rosetting have a reduced chance of developing severe falciparum malaria as compared to patients with other blood groups.

## Background

Malaria is caused by an obligate, intracellular protozoan parasite of the genus *Plasmodium*. Of the four species that infect humans (*Plasmodium falciparum*, *Plasmodium vivax, Plasmodium ovale *and *Plasmodium malariae*), *P*. *falciparum *is responsible for virtually all deaths [1]. The virulence of *P. falciparum *has been associated with the capacity of the infected RBCs to adhere to uninfected RBCs, leading to rosetting of cells [2,3]. Previous studies have implicated the ABO blood group type in rosetting [4]. Blood group antigens A and B are trisaccharides attached to a variety of glycoproteins and glycolipids on the surface of erythrocytes, and these trisaccharides are thought to act as receptors for rosetting on uninfected erythrocytes and bind to parasite rosetting ligands such as PfEMP-1 and sequestrin [5,6]. However, blood group antigens A and B are not expressed in blood group O individuals. As a result, rosettes formed by blood group O are suggested to be smaller and easily disrupted than rosettes formed by blood group A, B or AB erythrocytes [7,8]. The present study aimed to show whether blood group types are associated with the risk of severe *P*. *falciparum *malaria infection in three Ethiopian malaria endemic localities.

## Methods

### Study site

The study was conducted in Awash, Metehara and Ziway areas of Ethiopia. The study areas are characterized by seasonal malaria with a frequent occurrence of epidemics, often from September to December, following the heavy rainfall season. There is one malaria centre in each region where people with symptoms suggestive of malaria obtain free services for malaria diagnosis and treatment.

### Study participants

Patients attending the malaria centre at Ziway, Metehara and Awash were screened for malaria infection using thick blood film. *Plasmodium falciparum *malaria positive individuals who had received anti-malarial treatment within 48 hours prior to the microscopical confirmation of their malaria, patients critically ill and unable to respond for the interview and those co-infected with *P. falciparum *and other species of *Plasmodium *parasite were excluded from the study.

A total of 210 cases of malaria and 190 healthy controls participated in the study. Out of the 210 malaria patients, those who were positive for at least one of the three criteria of severe malaria (cerebral malaria, severe anaemia; haemoglobin concentration < 6 g/dl and circulatory collapse; systolic blood pressure < 80 mmHg in patients > 5 years of age, < 50 mmHg in children aged 1-5 years) as outlined by WHO [9] were included in the study.

### Laboratory analyses

#### Parasite density determination

Thick and thin blood film slides were prepared using 10% Giemsa solution. The stained slides were examined under a light microscope using 100 × oil immersions by an experienced laboratory technician. Parasitaemia was calculated per 200 white blood cells (WBC) assuming 8000 WBC/μl of blood [10].

#### Blood group determination

ABO blood groups were typed by agglutination using commercial antisera (Biotech laboratories Ltd, Ipswich, Suffolk, UK) [8,11]. Two drops of whole blood were placed in two different places of a grease-free clean glass slide on which a few drops of antisera for blood group A and B was applied. The blood cells and the antigen were mixed with applicator stick. The slide was then tilted to detect for agglutination and the result recorded accordingly [8,11].

#### Determination of haemoglobin concentration

Finger-prick samples were collected and haemoglobin measured by Hemocue™ (haemoglobinometer, Angelholm, Sweden).

### Statistical analysis

Data was analysed using SPSS software (version 13.0, Chicago, IL, USA) and SISA software. Chi-square (χ^2^) was used to determine association. Difference between means was analysed by ANOVA, and odds ratios (OR) were calculated with 95% confidence interval (CI). Values were considered to be statistically significant when P-values are less than or equal to 0.05.

### Ethical clearance

The study protocol was reviewed and approved by the Ethical Review Committee of Department of Biology, Addis Ababa University. Written informed consent was obtained from all study participants and mothers/caretakers of children under 18 who participated in the study after explaining the purpose and objective of the study.

## Results

### Characteristics of the study participants

Individuals suffering from most severe malaria symptoms were on average 14.3 years old and younger than individuals with uncomplicated malaria and healthy controls. However, there was no significant difference in mean age between the three groups (*P *= 0.80) (Table1). A significant difference was observed in haemoglobin concentration between the severe malaria (mean (± SD) 10. 4 (4.3) gm/dl)), uncomplicated malaria (mean (± SD) 12.0 (2.4) gm/dl) and healthy control cases (mean (± SD) 12.8 (2.41) gm/dl) (P < 0.0001) (Table1). Although the mean parasite density in individuals with severe malaria was higher than in those with uncomplicated malaria, the difference was not significant (P = 0.63) (Table 1).

**Table 1:** Characteristics of the study participants.

Category	Total (N)	Average age (years)	Mean Hb ***(g/dl *) **^**X**^	Mean Parasite density **per μl of blood **^**X**^
Severe malaria	70	14.3	10.4 (4.3)	3.44 (4.38)
Uncomplicated malaria	140	15.6	12.0 (2.4)	3.31 (4.67)
Healthy controls	190	15.0	12.8 (2.41)	NA^≠^
*P*- value^†^	NA^≠^	0.80	< 0.0001*	0.63

* Significant difference; ^X ^Values shown in bracket are the ± standard deviations; † ANOVA; NA^**≠**^, not applicable.

### Percentage distribution of the ABO blood group types in the three study categories

Of the 70 blood samples examined in the severe malaria category, there were 25 (35.7%), 15 (21.4%), 14 (20%) and 16 (22.9%) blood group A, B, AB and O patients, respectively. Among the uncomplicated malaria cases, 42 (30%) were of blood group A, 23 (16.4%) were of blood group B and 64 (45.7%) belonged to blood group O (Table 2).

**Table 2:** Percentage distribution of the ABO blood group types in the three study categories.

Category	**A **(%)	**B **(%)	**AB **(%)	**O **(%)
Severe malaria	25 (35.7)	15 (21.4)	14 (20.0)	16 (22.9)
Uncomplicated malaria	42 (30.0)	23 (16.4)	11 (7.9)	64 (45.7)
Healthy controls	53 (27.9)	35 (18.4)	23 (12.1)	79 (41.6)

There was low percentage of Blood group O patients in the severe malaria category than in either uncomplicated malaria or healthy controls. Blood group O made up only 22.9% of severe malaria patients compared to 45.7% and 41.6% of the uncomplicated and healthy controls, respectively (Table 2).

### The frequency of O and non-O blood group types between the three study categories

As compared to the uncomplicated malaria and healthy control cases, the case of severe malaria was more likely to be of type A (SM vs. UM: O vs. A, odds ratio (OR) 0.42, 95% confidence interval (CI) 0.20- 0.88; SM vs. HC: O vs. A, OR 0.43, 95% CI 0.21-0.88) and B (SM vs. UM: O vs. B, OR 0.38, 95% CI 0.16-0.89; SM vs. HC: O vs. B; OR 0.47, 95% CI 0.21-1.06) than type O. Furthermore, individuals with severe malaria were about six fold less likely to be of type O as to be of type AB (O vs. AB, odds ratio 0.19, 95% confidence interval 0.07-0.51) (Table 3).

**Table 3:** The odds ratios and P values for the frequency of O and non-O blood group types between the three study categories: patients with severe malaria (SM), uncomplicated malaria (UM), and healthy controls (HC).

Blood group compared	***SM vs. UM ***^**X**^	***SM vs. HC ***^**X**^	***UM vs. HC ***^**X**^
*O vs. A*	0.42,[0.20-0.88],(0.019)	0.43, [0.21-0.88],(0.02)	1.02,[0.60-1.72],(0.93)
*O vs. B*	0.38,[0.16-0.89],(0.02)	0.47,[0.21-1.06],(0.07)	1.23,[0.66-2.29 ],(0.50)
*O vs. AB*	0.19,[0.07-0.51],(0.0005)	0.33,[0.14-0.78],(0.009)	1.69, [0.76-3.73 ],(0.19)
*O vs. (A,B & AB)*	0.35,[0.18-0.67],(0.0013)	0.42,[0.22-0.78],(0.35)	1.18,[0.76-1.84],(0.45)

X values shown are the odds ratios, [95% CI], (P-value).

## Discussion

The demonstration that the cases of severe malaria were less likely to be of blood group O than other blood groups is in agreement with the report of Pathirana *et al *[1] from Sri Lanka, and the findings of this study substantiated earlier reports that blood group O individuals are less prone to come down with severe malaria as compared to those with other blood groups. The finding of greater infant length, placental weight and low placental parasite count among blood group O mother compared with non-group O in the Gambia [12] also supports the hypothesis that group O individuals may have survival advantage in severe falciparum malaria infection. Furthermore, according to study made on 489 malaria patients in Zimbabwe, comma was three times more common among group A individuals compared with non-A, and this study suggested that group O malaria patients tend to have mild disease and less clinical outcome than blood group A individuals [13]. The mechanism by which blood group O confers some protection against severe malaria compared to blood groups A, B, and AB is not fully understood. However, lower rosette formation by pRBCs of group O as shown by some studies [14,3] have established that parasitized erythrocytes form rosettes more readily with RBCs of either A, B, or AB blood groups than with those belonging to blood group O. Also, it is well established that this parasite-triggered RBC rosette formation is associated with the severity of clinical disease [3].

In the present study, there was high percentage of blood group A and low percentage of group O individuals in the severe malaria category than in the uncomplicated malaria and health controls. This was in consistence with the study in Sri Lanka [1], where there was low percentage of blood group O (23.8%) individuals as compared to the uncomplicated malaria (47.9%) and healthy controls (49.2%) and higher percentage of blood group A severe malaria (32.5%) patients. A study conducted on 200 malaria patients in Gabon also showed that, among all blood group A individuals, 71% had severe malaria and only 22% had mild malaria, and among all blood group O cases 46% had severe malaria and 54% had mild malaria [15].

In contrast to the observation of Rowe *et al *[16], the present study did not reveal individuals with severe malaria to have significantly higher parasite count than patients with uncomplicated malaria. This finding can be explained by the fact that in severe falciparum malaria infection, parasitized erythrocytes at schizont stage are known to be sequestered in tissue capillaries and may results in falsely low parasite count in the peripheral blood. Therefore, the severity of infection with falciparum malaria may have been greater in this study than that reported by Rowe *et al *[16].

In the present study, it was also been shown that individuals with severe malaria had lower haemoglobin concentration than those with uncomplicated malaria. Rowe *et al *[16] also provided a strong evidence in Mali that, venous blood haemoglobin concentration was lower in severe malaria patients than those with uncomplicated malaria or healthy controls. The basis for malaria mediated anaemia has been elucidated by several investigators [[2,16], and [17]]. Carlson *et al *[2] and Rowe *et al *[16] suggested that the virulence of *P. falciparum *is associated with the capacity of the infected RBC to adhere to uninfected RBC, a process known as rosetting. Hotez *et al *[17] also suggested that malaria cause anaemia by distraction of both parasitized and non-parasitized red blood cells. Therefore, the difference in mean haemoglobin concentration between the severe malaria and uncomplicated malaria or healthy control cases may be due to existance of increased distruction of red blood cells or higher rosetting in severe malaria patients.

The present study does not adjusted for the effect of other determinants such as genetic polymorphisms that might play key roles in the severity of falciparum infection, so that the effect of these factors could not be discussed in this paper.

## Conclusion

The study revealed that on the basis of the three criteria used to determine severity of falciparum malaria, patients with blood group O were less prone to severe malaria as compared to patients with other blood groups. However, since ABO blood group can also affect the severity of *P. falciparum *malaria by mechanisms other than rosetting, further studies of rosette formation with Ethiopian strains of *P. falciparum *could be useful to test the role of rosette formation in disease severity.

## Competing interests

The authors declare that they have no competing interests.

## Authors' contributions

ZT was involved in all aspects of the project, data collection, analysis, interpretation and determined haemoglobin concentrations and in writing of the manuscript. BP has made a contribution in the design, data interpretation, work supervision and in critically revising the manuscript. All authors read and approved the manuscript.
